# Developing effective and efficient study specific training to enable rapid study start up

**DOI:** 10.1186/1745-6215-14-S1-P129

**Published:** 2013-11-29

**Authors:** Lynda Constable, Alison McDonald, Cathryn Glazener

**Affiliations:** 1 University of Aberdeen, Aberdeen, UK

## 

Study specific training is an essential but often understated component of the study set up process for individual site personnel. One of the many challenges for the trial manager is to ensure study staff are appropriately trained without compromising rapid study start-up, quality of training, ethical compliance, GCP, running costs.

A variety of training methods exist with different studies employing one or more different training options. On site, centre specific (Site Initiation Visit (SIV/SV)), central, multi-centre (Investigator Meeting (IM)) or ‘virtual' IM training methods are often employed during start-up to enable in-depth protocol, safety, laboratory, informed consent and CRF completion guidelines and training. This ensures standardisation of study procedures across sites and that all site staff are informed of the procedures for conduct of the study.

GCP and research governance guidelines state all study personnel are ‘qualified by education, training and experience'. Training should be proportionate and tailored to the specific needs of the site/study personnel. It should promote GCP but not replace formal GCP training.

Experience from setting up a large UK multi-centre RCT (Vault or Uterine prolapse surgery Evaluatioin, VUE) indicates study sites appreciate the networking and open discussion opportunities provided by a central IM, but prefer the one to one site specific training that a SIV provides, and are particularly motivated by personal study training/information packs.

This presentation will discuss essential components of study training and different training methods to promote rapid trial start up as well as feedback from site personnel exposed to different training methods.

